# Assessing the viability of genebanked seeds from rare, wild plants native to the United States using the D.E.A.D. paradigm

**DOI:** 10.1002/aps3.70035

**Published:** 2025-12-30

**Authors:** Christina Walters, Lisa M. Hill, Katherine D. Heineman, Hannah M. Tetreault, Shaimaa Ibrahim, Katherine Markstein, Cheryl Birker, Kris Freitag, Dustin Wolkis, Michael Kunz, Sheila Murray, Nathaniel Kingsley, Alexandra Seglias, Nicholas Matsumoto, Thomas N. Kaye, Cheryl Peterson, Matthew A. Albrecht, Noah Dell, Stacy Anderson, Steve Blackwell, David Remucal, Wendy Gibble, Anita Tiller, Emily Coffey, Jason Ligon, Laurie Blackmore, Carrie Radcliffe, Heather E. Schneider, Kristen Nelson, David Sollenberger, Jessamine Finch, Kate Wellspring, Robert Jetton, Joyce Maschinski

**Affiliations:** ^1^ USDA‐ARS National Laboratory for Genetic Resources Preservation Fort Collins Colorado USA; ^2^ Center for Plant Conservation Escondido California USA; ^3^ San Diego Zoo Wildlife Alliance, Conservation Science Escondido California USA; ^4^ California Botanic Garden Claremont California USA; ^5^ Rae Selling Berry Seed Bank and Conservation Program Portland Oregon USA; ^6^ Department of Science and Conservation National Tropical Botanical Garden Kalāheo Hawaiʻi USA; ^7^ North Carolina Botanical Garden Chapel Hill North Carolina USA; ^8^ The Arboretum at Flagstaff Flagstaff Arizona USA; ^9^ University of Hawaiʻi Harold L. Lyon Arboretum Honolulu Hawaiʻi USA; ^10^ Denver Botanic Gardens Denver Colorado USA; ^11^ Fairchild Tropical Botanic Garden Coral Gables Florida USA; ^12^ Institute for Applied Ecology and Oregon State University Corvallis Oregon USA; ^13^ Bok Tower Gardens Lake Wales Florida USA; ^14^ Missouri Botanical Garden St. Louis Missouri USA; ^15^ Desert Botanical Garden Phoenix Arizona USA; ^16^ University of Minnesota Landscape Arboretum Minneapolis Minnesota USA; ^17^ University of Washington Botanic Gardens Seattle Washington USA; ^18^ Mercer Arboretum and Botanic Gardens Humble Texas USA; ^19^ Atlanta Botanical Garden Atlanta Georgia USA; ^20^ Santa Barbara Botanic Garden Santa Barbara California USA; ^21^ California Native Plant Society Sacramento California USA; ^22^ Chicago Botanic Garden Glencoe Illinois USA; ^23^ Native Plant Trust Wayland Massachusetts USA; ^24^ North Carolina State University Raleigh North Carolina USA

**Keywords:** genebank, germination, long‐term storage, rare plants, seed aging, seed banking, seed dormancy, seed longevity, seed storage, wild species

## Abstract

**Premise:**

Genebanks must maintain viable seeds for decades. Seeds that germinate are clearly alive, but some seeds, often from wild populations, do not germinate because they are dormant, empty, aged, or damaged (D.E.A.D.). This work evaluates the effects of D.E.A.D. factors on genebanked seeds using a unique dataset to improve genebanking practices and standards for ex situ conservation of seed collections.

**Methods:**

Seeds from over 100 species were recently collected from the same populations as seeds that were genebanked decades ago. Germination proportion and speed were measured after applying various temperature, chemical, or seed coat abrasion treatments. Viability was further tested using vital staining of samples with a low germination proportion. Proportions of dormant, empty, aged, and damaged seeds were compared between seed cohorts.

**Results:**

Germination proportion and speed varied among samples, and cues to stimulate germination of dormant seeds were identified for individual species, leading to a positive correlation between viability metrics of germination and vital staining. Empty seeds primarily contribute to low germination in this study. Aging, indicated by lower and slower germination, was evident in several of the stored samples, compared to those that had been recently harvested.

**Discussion:**

This unique approach demonstrates the feasibility of genebanking seeds from diverse endangered plant species using freezer storage. Genebanking methods that are more relevant for crop seeds need to be modified when applied to seeds from wild populations because the sample sizes tend to be small and the seeds tend to germinate slowly and asynchronously.

Seed testing is a basic research tool to understand seed biology and evaluate seed responses to imposed treatments. Seed testing also helps to predict field emergence and set market values for the seed trade (Powell and Matthews, 2012; AOSA, 2018). Genebanks also rely on seed testing to safeguard the future quality and utility of accessions submitted to scientific collections. At genebanks, tests of incoming seeds are used to document the sample size, verify taxon designation, account for inert components, and assess whether seed quality was harmed by growth or harvest conditions. Thus, performing germination tests on incoming seed accessions provides key insights that can guide future uses of seeds. Genebanks may repeatedly test stored seed samples to detect aging and anticipate the need to replenish a sample that may be approaching its life expectancy. This monitor testing is guided by best practices that seek to reduce genetic erosion within the genebank (FAO, 2014; MSBP, 2015; Etterson et al., 2016; CPC, 2019).

Seed tests for commercial, research, and most genebanking applications require a large number of uniformly behaving seeds to detect subtle differences in vigor that might reflect future field performance, response to a treatment, or degradation. Large sample sizes are required if results are presented as proportion data (e.g., normal vs. abnormal seedlings or alive vs. dead seeds), which have high variances that drive the statistical power of comparisons (AOSA, 2018; Tetreault et al., 2023). Crop seeds are ideal for laboratory testing because they are fecund and are bred for rapid, thorough, and synchronous germination. Increasingly, however, researchers, land managers, conservationists, and genebanking staff focus on seeds from wild populations because these genetic resources offer novel diversity and ecological benefits (Phillips and Meilleur, 1998; Merritt et al., 2014; MSBP, 2015; Greene et al., 2018; Khoury et al., 2020; Harrison et al., 2023). For many wild species, seed germination is slow (Baskin and Baskin, 2003, 2014), few seeds are available, and assays can be highly labor‐intensive and give variable results among replicates due to minor or unknown differences in testing conditions. Hence, genebanking seeds from wild sources presents new challenges to develop ways to process, store, and germinate small sample sizes of seeds from diverse species in a laboratory setting with little a priori information on the seed biology.

Testing wild seeds for initial and sustained quality may not follow the same paradigms that guide best practices for domesticated species. Currently, germination tests are the gold standard used for all seeds. Seed lots from freshly harvested crop species tend to germinate readily, whereas freshly harvested seeds from wild populations are subject to a host of constraints that affect germination and make growth requirements unpredictable. To gain insights on the reasons why many wild seeds do not germinate, we use the acronym D.E.A.D. to refer to seeds that may be dormant, empty, aged, or damaged. Factors such as seeds that lack embryos (i.e., unfilled or empty seeds) or have been damaged by insects, disease, or harvest practices are important for assessing the number of live seeds in a sample, but these factors are unlikely to affect the overall genebanking process or seed longevity (Mead and Gray, 1999).

Alternatives to germination assays might ameliorate problems associated with testing wild‐collected seeds, such as too few seeds being available, high variation among replicate seed tests, unknown germination requirements, and long, costly test durations. Tests indicating respiratory activity, such as positive staining with 2,3,5‐triphenyl tetrazolium chloride (TZ test), are frequently substituted for, or added to, a germination test when seeds are slow or fail to germinate (Copeland and McDonald, 2001; Riebkes et al., 2015). In other words, TZ tests are frequently used to distinguish between seeds that are alive and dormant versus those that are inviable (Miller, 2005; AOSA/SCST, 2010). Emerging techniques that quantify respiratory capacity via oxygen consumption may have more predictive power than binary (i.e., alive/dead) characterizations (Bello and Bradford, 2016; Dalziell and Tomlinson, 2017; Bajerski et al., 2018). Tests indicating membrane integrity, including osmotic responsiveness and electrolyte leakage, have also been favored as proxies for viability (Powell and Matthews, 2012; Volk and Caspersen, 2017; Marin et al., 2018; Kovaleski and Grossman, 2021). We are currently exploring tests that reflect RNA integrity (Tetreault et al., 2025) and gene expression patterns (Fleming et al., 2021) to quantify the nature and kinetics of seed deterioration as well as the prognosis for recovery following long‐term storage (Fleming et al., 2019; Tetreault et al., 2024). To resolve problems intrinsic to testing wild seeds, alternative tests should use methods that can be standardized across a wide range of species and, ideally, are adaptable to automation.

In addition to alternative viability indicators, stress tests are designed to characterize seed vigor or health. These tests originated under an assumption that an unhealthy, but viable, seed will not survive stressful germination conditions, such as low temperature or water availability (Powell and Matthews, 2012). Stress tests have since evolved to be indicators of seed longevity, based on the idea that stressed seeds will succumb before unstressed seeds or that deterioration rates at high humidity and temperature (i.e., “accelerated aging”) correlate with rates at low humidity and temperature (Rajjou et al., 2008; Powell and Matthews, 2012; Hay et al., 2019). Numerous studies have compared seed longevities among wild species using accelerated aging approaches (Mondoni et al., 2011; Nguyen et al., 2012; Merritt et al., 2014; Franks et al., 2019; Davies et al., 2020; Niñoles et al., 2022). Unfortunately, there are few guidelines to relate survival times at elevated relative humidity and temperatures to the longevity achieved by genebanks (Niedzielski et al., 2009; Walters et al., 2020). Hence, there is a great need for empirical data to document how well genebanking conditions preserve seed viability. These data are increasingly available for crop seeds that have been stored in some genebanks since the 1960s and 1970s (Walters et al., 2005; Pérez‐García et al., 2007); however, there are fewer reports of wild seed survival in genebanks. This dearth of information is partly attributed to the challenges of testing wild seeds, as described above. In addition, data related to the survival of wild seeds placed in long‐term storage are relatively rare because initiatives to genebank seeds from the wild are relatively recent (MSBP, 2015; Etterson et al., 2016; Harrison et al., 2023) with a few notable exceptions (Pérez‐García et al., 2007; Kennedy, 2008; Wolkis and Deans, 2019).

This paper is part of a series exploring the efficacy of genebanking seeds from wild species that are of conservation concern within the United States (see also Tetreault et al., 2025). Efficacy is roughly defined as whether or how long seeds survived. Here, we examine the germination behavior of over 100 species that are included in the Center for Plant Conservation's (CPC) National Collection, which was initiated in 1984 (Kennedy, 2008). The samples used in this study were placed into storage between 1983 and 2010 (i.e., stored cohorts). The viability of those accessions was not assessed until now because past priorities likely emphasized making collections, or at least not prematurely depleting samples; however, concern for lost viability in these stored seeds now prompts an assessment. To this end, a second set of seeds were harvested in 2021–2024 from the same populations as the stored cohorts. These recently harvested cohorts provide a resource to assess viability and germination patterns without the confounding factor of decades of storage. Using both cohorts and applying the D.E.A.D. paradigm, we examine the initial quality of wild seeds as well as how these factors may contribute to genebanking success. We categorize species by the speed at which seeds germinate and by the pretreatments that stimulate (or retard) germination. In subsequent reports, we will use the data provided here to develop alternative methods to document seed survival and longevity. Overall, this paper provides a rich assessment of germination patterns for a diverse set of species with the overall goal of enhancing efforts to bank genetic resources of wild plant species.

## METHODS

### Plant materials

Seeds from over 100 species were collected by botanical experts from CPC Participating Institutions (Tables 1, 2). Species were selected to represent diverse habitats, life history traits, and botanical families across the United States. The original collections were made between 1983 and 2010 (Table 2), and most of the samples were stored according to Food and Agriculture Organization of the United Nations (FAO) standards of dry (relative humidity between 15% and 25%) and cold (−18°C) conditions (FAO, 2014). Seeds from a few species were stored under refrigerated (5°C) or cryogenic (approximately −180°C) conditions. Seeds from the same populations, or founder individuals from those populations growing ex situ, were recollected between 2021 and 2024, and the assessed quality of these recently harvested seeds served as the primary reference for initial seed quality. The number of seeds available for testing often varied by seed size, with many seeds available for tiny seeds (<0.08 mg/seed) and fewer seeds available for moderate and large seeds (>0.8 mg/seed). We set an initial goal of consuming <100 seeds per species per cohort to develop and assess germination behavior for these endangered species, allowing an additional 25–30 seeds to conduct assessments of respiratory capacity using a vital stain (described below) (Table 3).

**Table 1:** Center for Plant Conservation Participating Institutions that provided botanical expertise and collected the seeds used in this study.

Institution	Code	Location
Atlanta Botanical Garden	ABG	Atlanta, Georgia, USA
Rae Selling Berry Seed Bank and Conservation Program	BERR	Portland, Oregon, USA
Bok Tower Gardens	BOK	Lake Wales, Florida, USA
California Botanic Garden	CalBG	Claremont, California, USA
Camcore, North Carolina State University	CAMCORE	Raleigh, North Carolina, USA
Chicago Botanic Garden	CBG	Glencoe, Illinois, USA
California Native Plant Society	CNPS	Sacramento, California, USA
Denver Botanic Gardens	DBG	Denver, Colorado, USA
Desert Botanical Garden	DES	Phoenix, Arizona, USA
The Arboretum at Flagstaff	FLAG	Flagstaff, Arizona, USA
Fairchild Tropical Botanic Garden	FTBG	Coral Gables, Florida, USA
Harold L. Lyon Arboretum	HLA	Honolulu, Hawaii, USA
Institute for Applied Ecology	IAE	Corvallis, Oregon, USA
Missouri Botanical Garden	MBG	St. Louis, Missouri, USA
Mercer Arboretum and Botanic Gardens	MERC	Humble, Texas, USA
North Carolina Botanical Garden	NCBG	Chapel Hill, North Carolina, USA
National Laboratory for Genetic Resources Preservation	NLGRP	Fort Collins, Colorado, USA
Native Plant Trust	NPT	Wayland, Massachusetts, USA
National Tropical Botanical Garden	NTBG	Kalaheo, Hawaii, USA
Santa Barbara Botanic Garden	SBBG	Santa Barbara, California, USA
San Diego Zoo Wildlife Alliance	SDZWA	San Diego, California, USA
University of Minnesota Landscape Arboretum	UMLA	Minneapolis, Minnesota, USA
University of Washington Botanic Gardens	UWBG	Seattle, Washington, USA

**Table 2:** The species that were included in this study, the abbreviation used to identify them, harvest years for stored and recently harvested cohorts, and the responsible institution for identifying the plants, processing the seeds, and in many cases genebanking the accession at −18°C. A key to the donating institutions is provided in Table 1.

Taxon and synonym	Authority	Species code	Botanical family	Donating institution (recent harvest)	Harvest year	Donating institution (stored seed)	Harvest year
*Abies fraseri*	(Pursh) Poir.	abfr	Pinaceae	CAMCORE	2023	CAMCORE	2006
*Abronia umbellata* subsp*. breviflora*	(Standl.) Munz	abum	Nyctaginaceae	IAE	2021	BERR	1990
*Actaea arizonica*	(S. Watson) J. Compton	acar	Ranunculaceae	FLAG	2022	FLAG	1994
*Agalinis densiflora*	(Benth.) S.F. Blake	agde	Orobanchaceae	CBG	2021	CBG	1997
*Aletes humilis*	J.M. Coult. & Rose	alhu	Apiaceae	DBG	2021	DBG	1988
*Amaranthus pumilus*	Raf.	ampu	Amaranthaceae	NCBG	2022	NCBG	1987
*Amelanchier nantucketensis*	E.P. Bicknell	amna	Rosaceae	NPT	2023	NPT	1993
*Amorpha herbacea* var*. crenulata*	(Rydb.) Isely	amhe	Fabaceae	FTBG	2021	FTBG	2003
*Amsonia tharpii*	Woodson	amth	Apocynaceae	DES	2023	DES	1989
*Anemone patens* var*. multifida*	Pritz.	anpa	Ranunculaceae	UWBG	2021	UWBG	1993
*Arctostaphylos catalinae*	P.V. Wells	arca	Ericaceae	SBBG	2023	CalBG	1995
*Argemone glauca*	Pope	argl	Papaveraceae	NTBG	2022	NTBG	1996
*Aster furcatus* or *Eurybia furcata*	E.S. Burgess or (E.S. Burgess) G.L. Nesom	asfu	Asteraceae	CBG	2021	CBG	1998
*Astragalus albens*	Greene	asal	Fabaceae	CalBG	2021	CalBG	1995
*Astragalus bibullatus*	Barneby & E.L. Bridges	asbi	Fabaceae	MBG	2021	MBG	1993
*Astragalus linifolius* or *Astragalus rafaelensis*	Osterh. or M.E. Jones	asli	Fabaceae	DBG	2023	DBG	1987
*Astragalus magdalenae* var*. peirsonii*	(Muna & McBurney) Barneby	asma	Fabaceae	CalBG	2021	CalBG	2003
*Astragalus tegetarioides*	M.E. Jones	aste	Fabaceae			BERR	1999
*Astragalus tyghensis*	M. Peck	asty	Fabaceae	BERR	2022	BERR	2000
*Berberis nevinii*	A. Gray	bene	Berberidaceae	CalBG	2021	CalBG	1990
*Besseya bullii*	(Eaton) Rydb.	bebu	Scrophulariaceae	UMLA	2022	CBG	1986
*Bidens torta*	Sherff	bito	Asteraceae	HLA	2022	HLA	2000
*Boechera parishii* or *Arabis parishii*	(S. Watson) Al‐Shehbaz or S. Watson	bopa	Brassicaceae	CalBG	2021	CalBG	1991
*Bromus carinatus* var*. carinatus*	Hook. & Arn.	brca	Poaceae	SDZWA	2024	SDZWA	2005
*Calochortus umpquaensis*	Fredericks	caum	Liliaceae	BERR	2022	BERR	1993
*Campanula scabrella*	Engelm.	casc	Campanulaceae	CNPS	2023	CalBG	1994
*Carex comosa*	Boott	caco	Cyperaceae	UWBG	2022	UWBG	2003
*Carex oronensis*	Fernald	caor	Cyperaceae	NPT	2024	NPT	1991
*Castela emoryi*	(A. Gray) Moran & Felger	caem	Simaroubaceae	CBG	2021	CBG	2004
*Castilleja kaibabensis*	N.H. Holmgren	caka	Orobanchaceae	FLAG	2021	FLAG	1989
*Ceanothus cyaneus*	Eastw.	cecy	Rhamnaceae	SDZWA	2021	CalBG	1990
*Chenopodium oahuense*	Aellen	choa	Amaranthaceae	NTBG	2022	NTBG	1998
*Chrysopsis floridana*	Small	chfl	Asteraceae	BOK	2021	BOK	1989
*Cimicifuga elata*	Nutt.	ciel	Ranunculaceae	IAE	2022	BERR	1994
*Cirsium pitcheri*	Torr. & A. Gray	cipi	Asteraceae	CBG	2021	CBG	1991
*Clarkia biloba* subsp*. australis*	F.H. Lewis & M.E. Lewis	clbi	Onagraceae	CNPS	2023	CalBG	1991
*Clematis socialis*	Kral	clso	Ranunculaceae	ABG	2021	NCBG	1993
*Clermontia kakeana*	Meyen	clka	Campanulaceae	HLA	2021	HLA	1997
*Cordylanthus maritimus* subsp*. palustris*	(Behr) T.I. Chuang & Heckard	coma	Orobanchaceae	IAE	2021	BERR	1990
*Cyanea angustifolia*	(Cham.) Hillebr.	cyan	Campanulaceae	HLA	2021	HLA	1997
*Cyperus javanicus*	Houtt.	cyja	Cyperaceae	NTBG	2022	NTBG	2008
*Dalea foliosa*	(A. Gray) Barneby	dafo	Fabaceae	MBG	2022	MBG	2000
*Deinandra increscens* subsp*. villosa*	(Tanowitz) B.G. Baldwin	dein	Asteraceae	SBBG	2021	SBBG	2003
*Deinandra mohavensis*	(D.D. Keck) B.G. Baldwin	demo	Asteraceae	CalBG	2021	CalBG	2002
*Dicerandra immaculata*	Lakela	diim	Lamiaceae	BOK	2022	BOK	1987
*Dodonaea viscosa*	Jacq.	dovi	Sapindaceae	NTBG	2022	NTBG	1990
*Dubautia menziesii*	D.D. Keck	dume	Asteraceae	HLA	2021	HLA	2002
*Echinacea tennesseensis*	(Beadle) Small	ecte	Asteraceae	MBG	2021	MBG	1994
*Echinocactus horizonthalonius* var*. nicholii*	L.D. Benson	echo	Cactaceae	DES	2022	DES	1991
*Erigeron parishii*	A. Gray	erpa	Asteraceae	CalBG	2021	CalBG	1991
*Eriogonum crosbyae*	Reveal	ercr	Polygonaceae			BERR	1983
*Eriogonum cusickii*	M.E. Jones	ercu	Polygonaceae	BERR	2022	BERR	1983
*Eryngium aristulatum* var*. parishii*	(J.M. Coult. & Rose) Mathias & Constance	erar	Apiaceae	SDZWA	2022	CalBG	1990
*Eurybia furcata* or *Aster furcatus*	(E.S. Burgess) G.L. Nesom or E.S. Burgess	eufu	Asteraceae	CBG	2021	CBG	1998
*Eustachys petraea*	(Sw.) Desv.	eup3	Poaceae	FTBG	2024	FTBG	2009
*Eutrema penlandii* or *Eutrema edwardsii*	Rollins or R. Br.	eupe	Brassicaceae	DBG	2022	DBG	1988
*Gentiana newberryi*	A. Gray	gene	Gentianaceae	BERR	2023	BERR	1994
*Geum geniculatum*	Michx.	gege	Rosaceae	NCBG	2021	NCBG	1988
*Gilia leptantha* subsp*. leptantha*	Parish	gile	Polemoniaceae	CalBG	2022	CalBG	2003
*Hedeoma diffusa*	Greene	hedi	Lamiaceae	FLAG	2021	FLAG	1988
*Helonias bullata*	L.	hebu	Melanthiaceae	ABG	2024	NCBG	1991
*Hesperocyparis forbesii* or *Cupressus guadalupensis* var*. forbesii*	(Jeps.) Bartel or (Jeps.) Little	hefo	Cupressaceae	SDZWA	2021	CalBG	1995
*Hibiscus dasycalyx*	S.F. Blake & Shiller	hida	Malvaceae	MERC	2021	MERC	1993
*Horkelia hendersonii*	Howell	hohe	Rosaceae	BERR	2021	BERR	1989
*Hymenoxys texana*	(J.M. Coult. & Rose) Cockerell	hyte	Asteraceae	MERC	2021	MERC	2005
*Kalmiopsis fragrans*	Meinke & Kaye	kafr	Ericaceae	BERR	2021	BERR	2003
*Leiophyllum buxifolium* or *Kalmia buxifolia*	(Bergius) Elliott or (Bergius) Gift & Kron	lebu	Ericaceae	NCBG	2021	NCBG	1993
*Liatris novae‐angliae*	(Lunell) Shinners	lino	Asteraceae			NPT	1991
*Lilium parryi*	S. Watson	lipa	Liliaceae	CalBG	2021	CalBG	1990
*Linum carteri* var*. carteri*	Small	lica	Linaceae	FTBG	2021	FTBG	2003
*Lomatium bradshawii*	(Rose ex Mathias) Mathias & Constance	lobr	Apiaceae	IAE	2021	BERR	1990
*Lupinus westianus* var*. aridorum*	(McFarlin ex Beckner) Isely	luwe	Fabaceae	BOK	2021	BOK	2010
*Lycium sandwicense*	A. Gray	lysa	Solanaceae	NTBG	2021	NTBG	2006
*Metrosideros polymorpha* var*. polymorpha*	Gaudich.	mepo	Myrtaceae	HLA	2022	HLA	1999
*Muhlenbergia microsperma*	(DC.) Kunth	mumi	Poaceae	SDZWA	2024	SDZWA	2010
*Nolina brittoniana*	Nash	nobr	Agavaceae	BOK	2021	BOK	1986
*Ornithostaphylos oppositifolia*	(Parry) Small	orop	Ericaceae	SDZWA	2022	CalBG	1991
*Osteomeles anthyllidifolia*	(Sm.) Lindl.	osan	Rosaceae	HLA	2021	HLA	2000
*Oxypolis canbyi*	(J.M. Coult. & Rose) Fernald	oxca	Apiaceae	NCBG	2022	NCBG	1988
*Packera franciscana* or *Senecio franciscanus*	(Greene) W.A. Weber & Á. Löve or Greene	pafr	Asteraceae	FLAG	2022	FLAG	1991
*Penstemon clutei*	A. Nelson	pecl	Scrophulariaceae	FLAG	2021	FLAG	1991
*Penstemon peckii*	Pennell	pepe	Scrophulariaceae	BERR	2022	BERR	1992
*Penstemon shastensis*	D.D. Keck	pesh	Scrophulariaceae	CNPS	2023	CalBG	1993
*Phacelia formosula*	Osterh.	phfo	Boraginaceae	DBG	2021	DBG	1987
*Physaria globosa*	(Desv.) O'Kane & Al‐Shehbaz	phgl	Brassicaceae	MBG	2021	MBG	1995
*Physaria obcordata*	Rollins	phob	Brassicaceae	DBG	2022	DBG	1987
*Pinus radiata*	D. Don	pira	Pinaceae			NLGRP	2005
*Pityopsis ruthii*	(Small) Small	piru	Asteraceae	NCBG	2022	NCBG	1994
*Plagiobothrys hirtus*	(Greene) I.M. Johnst.	plhi	Boraginaceae	IAE	2021	BERR	1987
*Polemonium eddyense* or *Polemonium chartaceum*	Stubbs or H. Mason	poed	Polemoniaceae	CNPS	2019	NLGRP	1991
*Polemonium occidentale* subsp*. lacustre*	Wherry	pooc	Polemoniaceae	UMLA	2021	CBG	1998
*Polyscias racemosa* or *Polyscias lallanii*	(Drake) R. Vig. or R.Kr. Singh & Sanjeet Kumar	pora	Araliaceae	NTBG	2021	NTBG	1999
*Ptilimnium nodosum*	(Rose) Mathias	ptno	Apiaceae	NCBG	2022	NCBG	1987
*Purshia subintegra*	(Kearney) Henrickson	pusu	Rosaceae	FLAG	2021	FLAG	1998
*Remirea maritima*	Aubl.	rema	Cyperaceae	FTBG	2021	FTBG	2003
*Remya kauaiensis*	Hillebr.	reka	Asteraceae	NTBG	2022	NTBG	1990
*Rhus kearneyi* subsp. *kearneyi*	F.A. Barkley	rhke	Anacardiaceae	DES	2022	DES	1986
*Sarracenia oreophila*	Wherry	saor	Sarraceniaceae	ABG	2021	NCBG	1987
*Schoenoplectus tabernaemontani*	(C.C. Gmel.) Palla	scta	Cyperaceae	NTBG	2021	NTBG	2005
*Senecio ertterae*	T.M. Barkley	seer	Asteraceae	IAE	2021	BERR	1994
*Sesbania tomentosa*	Hook. & Arn.	seto	Fabaceae	NTBG	2021	NTBG	1996
*Sidalcea nelsoniana*	Piper	sine	Malvaceae	IAE	2021	BERR	1985
*Sisyrinchium sarmentosum*	Suksd. ex Greene	sisa	Iridaceae	BERR	2023	BERR	1996
*Solidago plumosa*	Small	sopl	Asteraceae	NCBG	2021	NCBG	2003
*Tephrosia angustissima* var. *corallicola*	(Small) Isely	tean	Fabaceae	FTBG	2022	FTBG	2000
*Vaccinium boreale*	I.V. Hall & Aalders	vabo	Ericaceae	NPT	2023	NPT	1997
*Vaccinium crassifolium* subsp. *sempervirens*	(D.A. Rayner & J. Hend.) W.B. Kirkman & Ballingt.	vacr	Ericaceae	NCBG	2021	NCBG	1988
*Warea amplexifolia*	(Nutt.) Nutt.	waam	Brassicaceae	BOK	2021	BOK	1988
*Ziziphus celata*	Judd & D.W. Hall	zice	Rhamnaceae	BOK	2021	BOK	2008

**Table 3:** General information about seed germination and viability results obtained in this study. Values are given as count data or average ± standard deviation among all species listed in Table 2.

Plant materials	Recently harvested cohort	Stored cohort	Related population
No. of families	39	40	15
No. of species	105	108	15
Harvest year	2021–2024	1983–2010	1985–2017
**Germination assays**			
Average no. of germinating assays/species (i.e., replicates)	2.8 ± 1.5	2.6 ± 1.5	2 ± 0.8
No. of seeds sown per species/age	98 ± 62	94 ± 61	78 ± 48
No. of seeds per assay	37 ± 36	36 ± 12	39 ± 17
Duration of assay (days)	164 ± 126	183 ± 154	129 ± 55
Time to reach 67% of final germination proportion (days)	74 ± 87	81 ± 87	56 ± 46
Average germination proportion	0.66 ± 0.29	0.50 ± 0.36	0.43 ± 0.39
Average proportion of unfilled seeds	0.19 ± 0.24	0.20 ± 0.24	0.22 ± 0.27
**Vital stain assays**			
No. of species assayed	59	59	7
No. of assays per species	1.4	1.4	1.2
Average proportion of positive staining in TZ tests	0.57 ± 0.28	0.50 ± 0.32	0.34 ± 0.30
Average germination proportion for TZ‐tested seeds	0.53 ± 0.29	0.39 ± 0.36	0.30 ± 0.39

In a few cases, we had access to additional seeds that were harvested from nearby populations in the years between the stored and recently harvested cohorts. These additional samples provided a valuable resource to confirm both germination testing methods and results, as well as to probe the effects of shorter storage durations.

Sometimes, conspecific or congeneric seeds were commercially available (sourced from Sheffield's Seed Company, Locke, New York, USA; Everwilde Farms, Fallbrook, California, USA; American Meadows, Shelburne, Vermont, USA; High Country Gardens, Clinton, Utah, USA) or included in the inventories of the National Plant Germplasm System (NPGS) (https://www.ars‐grin.gov/npgs/), and we accessed these seeds to experiment with germination treatments before working with seeds from the endangered species included in this study (data not presented).

### Germination assays

Germination protocols provide details for substrata, hydration solution (water or a solution of gibberellic acid [GA_3_], potassium nitrate [KNO_3_], or plant preservative mixture [PPM]; Plant Cell Technology, Washington, D.C., USA), germination temperature, and pretreatments (e.g., stratification or scarification) that maximize germination proportion and minimize assay duration (AOSA, 2018). This type of information is currently unavailable for nearly all of the species included in this study; however, information about dormancy mechanisms and dormancy‐breaking treatments is available for congeners of most of our study species (Deno, 1993; Baskin and Baskin, 2014). Initial treatments were guided by the experiences of the coauthors and National Laboratory for Genetic Resources Preservation (NLGRP) staff for related taxa, as well as by the CPC's database, climate information during seed maturation and seedling emergence seasons, and literature searches about germination behavior in similar habitats (e.g., Baskin and Baskin, 2014). Our assays initially favored the use of alternating daily temperatures of 15/25°C or 20/30°C (12 h each, with light delivered during the higher temperature). If sufficient seed numbers were available for subsequent tests, we tested the effects of a constant incubation temperature midrange from the alternating temperature range (i.e., 20°C and 25°C, respectively).

The number of seeds sown in a single assay ranged from 14 to 80, depending on seed availability and size (averaging about 36; Table 3). We usually sowed seeds on blue blotter paper (Anchor Paper Company, St. Paul, Minnesota, USA) in Petri plates, hydrated with deionized water or 0.2% PPM. We used sphagnum moss (from Kapecute, Guangzhou, China or Sun Gro Horticulture, Agawan, Massachusetts, USA) as the substrata if large seeds (>20 mg/seed) tended to mold or germinate slowly, as this helped to retain moisture without encouraging fungal growth. Depending on seed availability, between one and seven assays were conducted per species × cohort group (averaging about 2.7 among species × cohort combinations; Table 3) and between 17 and 385 seeds were consumed by germination testing (averaging 91 seeds per species × cohort group; Table 3).

Radicle emergence was counted daily to monthly, depending on temperature (lower frequency at lower temperatures) and other factors (anticipated germination speed, treatments to induce germination, and observations from earlier assays). We aimed for five to 10 observations to develop germination time courses used to calculate germination speed. If a sample fully germinated within a single observation interval, the assay was repeated and observations were made more frequently. The assay was deemed complete when all of the seeds either germinated or lost physical integrity (became squishy or moldy), which ranged from three to over 800 days. Seeds that appeared to be dead were dissected to determine if they were hollow or lacked an embryo (referred to as empty).

We calculated germination proportion for the assay as the total number of germinated seeds divided by the total number of sowed seeds. We often exposed seeds to different conditions in different assays, and therefore it may be inappropriate to call these replicates per se. For each species × cohort combination, we identified the assay that resulted in the highest germination proportion. Then, results from other assays were combined to ensure germination results were based on more than 20 seeds or included assays with similar outcomes (i.e., germination proportion was within 0.2 of maximum). Combining results among treatments/assays was done by summing the total number of germinating and sown seeds among the assays (Crawley, 2012). For example, if two assays of a sample yielded germination proportions of 0.90 and 0.75 (30 and 16 sown seeds, respectively), the reported germination proportion is 0.85 (0.848 = [27 + 12] ÷ [30 + 16]). The total number of seeds used for the reported germination proportions ranged from 20 (*Astragalus albens*, *Castela emoryi*, and *Osteomeles anthyllidifolia*; authorities for all study taxa are provided in Table 2) to over 200 (*Abies fraseri*, *Kalmiopsis fragrans*, or *Metrosideros polymorpha* var. *polymorpha*; large seed numbers are due to a high proportion of empty seeds [*Abies fraseri* and *Metrosideros polymorpha*] or tiny seeds [*Kalmiopsis fragrans*, 0.012 mg/seed]), with the average number of seeds used to report achieved germination proportions being 66.

Germination speed was calculated for all of the ~600 assays conducted in the study (i.e., 105 species × 2 cohorts × 2.8 germination assays) and is expressed as the incubation time over which seeds germinate (i.e., days after sowing). We used the time required for 67% (two‐thirds) of the final number of germinating seeds in each assay, reasoning that this best represents the sample without biasing the measurement with long durations for a few remaining seeds that eventually germinated. Germination speed was calculated from the date at which the germination proportion reached 0.67; for example, if 24 out of 30 seeds germinated in an assay, we identified the date at which 16 (67% of 24) of the seeds germinated, and this date was then subtracted from the sow date to give the days to 67% germination. Germination speed among multiple assays was averaged when the germination proportion data were combined. If no seeds germinated in a particular assay, the germination speed was not calculated.

### Dormancy‐breaking treatments

Seeds from approximately one‐fourth of the species in this study exhibit no other germination requirements than adequate moisture and warmth. For most of the species studied, seed treatments to stimulate germination involved combinations of stratification (hydrated storage at cool [5°C] temperatures), scarification (clipping, abrading, or peeling outer seed layers to increase water or oxygen permeation), exposure to dormancy‐breaking chemicals (GA_3_ or dilute KNO_3_ solutions), or heat shock (exposure to >35°C with or without water for a brief period) (Deno, 1993; Baskin and Baskin, 2014). We selected from numerous possible approaches based on institutional experience and literature reviews (e.g., Deno, 1993; Baskin and Baskin, 2014; and online sources) and tracked the effectiveness of various treatments by measuring the number of seeds that germinated before and after a dormancy‐breaking treatment.

Stratifying seeds upon sowing was our preferred approach for seeds that tend to overwinter and sprout in spring or if congeners express physiological dormancy (Baskin and Baskin, 2003, 2014). The duration of 5°C exposure was initially determined by the duration of winters at the latitude of the source population—one month for subtropical areas and up to four months for the northernmost populations of the species. Following the low temperature treatment, seeds were placed at warm temperatures (15/25°C, 20/30°C, 20°C, or 25°C) based on climate data for seedling emergence at their source locale. After switching from cold to warmth, seeds were observed frequently for germination (usually once or twice a week). We used the “move‐along” approach to cycle seeds through cool and warm treatments described by Baskin and Baskin (2003), which sometimes involved moving samples that showed no signs of germination during the initial incubation at warmer temperatures to 5°C conditions.

The need for scarification to increase water permeability was obvious if seeds did not swell or soften. For seeds from Fabaceae or Malvaceae, we usually made a thin (<0.5 mm) slice distal to the hilum and embryonic axis using a scalpel or straight‐edge razor blade. In slow‐to‐germinate seeds of Asteraceae and large seeds of Ericaceae species, we peeled or scraped the outer coverings using a scalpel a few months after sowing the seeds.

Solutions of GA_3_ (0.02%) (PhytoTech Labs, Lenexa, Kansas, USA) or KNO_3_ (0.2%) (Thermo Fisher Scientific, Waltham, Massachusetts, USA), rather than deionized water or PPM, were used for initial hydration of seeds or during watering treatments in assays extending over multiple months. Usually, the solutions were pipetted onto the blue blotter paper, but occasionally the seeds were soaked in solutions overnight before sowing. GA_3_ effects were tested in Brassicaceae, Poaceae, and seeds that appeared unresponsive to stratification or were from latitudes with relatively short, warm winters. For species with limited seed numbers, GA_3_ was often delivered in combination with stratification or scarification treatments, while the effect of GA_3_ alone was tested if there were sufficient numbers of seeds.

We restricted heat shock treatments to seeds originating from tropical or subtropical areas that also contain storage lipids with melting temperatures >10°C (Crane et al., 2003; Walters et al., unpublished). Seeds were exposed to daily alternating temperatures of 25/35°C for about a week after sowing.

### Vital staining

We used 1% 2,3,5‐triphenyl tetrazolium chloride (TTC) stain (Alfa Aesar, Thermo Fisher Scientific) to assess viability in low‐ or slow‐germinating samples. About 25–30 seeds were hydrated on blotter paper with deionized water for 24–48 h. Hydrated seeds were cut with a sharp edge or pierced with a sewing needle, and empty seeds were removed and counted. Filled seeds were submerged in the TTC solution for 8–48 h depending on the species and speed of color change. We assessed the pattern of red staining (embryo vs. nutritive tissues), as well as the speed of color development, based on the standard recommendations for botanical families (Miller, 2005; AOSA/SCST, 2010).

### Calculations and statistical analyses

We calculated descriptive statistics, compared means (*t*‐test of means using paired samples), and tested for correlation using the Analysis ToolPak for statistical functions in Excel (Microsoft Corporation, Redmond, Washington, USA).

## RESULTS

Over 600 germination assays and 160 TZ tests were performed on seeds from over 100 species (40 botanical families) from cohorts that were recently harvested (between 2021 and 2024) or stored for 28 ± 7 years at −18°C (harvested between 1983 and 2010) (Table 3). Additionally, seeds from 14 species from related populations harvested between 1985 to 2017 (mean year = 2002) were assayed to provide additional information on germination behavior or intermediate storage times. To provide ample time for seeds to germinate or decompose, germination assays tended to be long, continuing for 164 ± 127 (recent cohort) or 183 ± 154 (stored cohort) days on average, which can be expected when germination requirements are unknown (Table 3).

### Viability and germination speed of recently harvested seed samples

**Figure 1:**
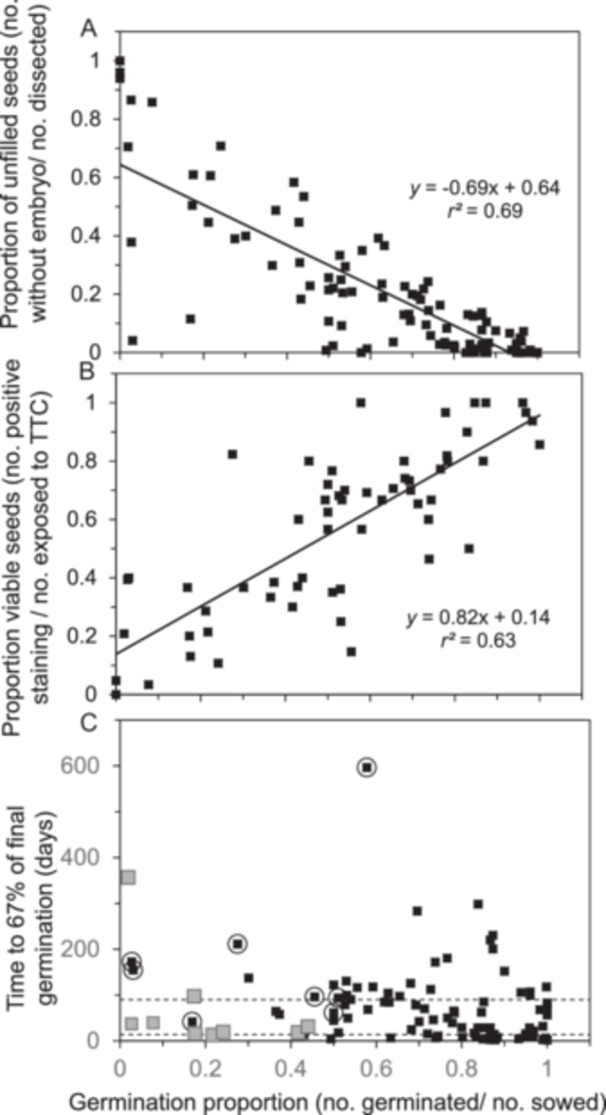
Relationships between various measurements of viability and vigor in recently harvested seeds from 105 species. The germination proportion negatively correlates with the proportion of empty seeds (A) and positively correlates with respiratory activity determined using staining patterns of a TZ test (B). Germination proportion does not correlate with germination speed, which is an indicator of dormancy (C). In C, gray points represent samples with a high proportion of empty seeds (>0.5) and encircled points represent samples with unbroken dormancy.

Germination behavior varied widely among the 105 seed samples in the recently harvested cohort. The proportions of germinating seeds ranged from 0 to 1, averaged (± SD) 0.66 ± 0.29 (Table 3), and were negatively correlated with the proportion of empty seeds (Figure 1A; *F* = 224, *df* = 103, *P* « 0.01). Based on *r* ^2^ = 0.69, we can conclude that seeds lacking embryos contributed to more than two‐thirds of the variation in germination potential observed within this study set. More than half of the seeds were empty in 12 samples, which explains their low germination proportions (points in the upper left portion of Figure 1A, see Table 4 for data on individual species). More puzzling were the samples with high seed fill but low germination proportion (points in the lower left portion of Figure 1A). These seeds could be either viable (dormant) or inviable (aged or damaged). To distinguish these possibilities, we further characterized viability using TZ tests if one or both cohorts exhibited low or slow germination. Accordingly, the average germination of TZ‐tested samples was lower than the overall averages of the cohort (0.53 ± 0.27, *n* = 59) (Table 3); however, the two methods of quantifying viability were positively correlated (Figure 1B; *F* = 100, *df* = 57, *P* « 0.01). Unbroken dormancy was presumed in seeds of *Actaea arizonica*, *Arctostaphylos catalinae*, *Cimicifuga elata*, *Cirsium pitcher*, *Clematis socialis*, *Oxypolis canbyi*, and *Sisyrinchium sarmentosum* because the difference in proportions of positive TZ test staining and germinating seeds exceeded 0.2. Seeds of *Polemonium occidentale* subsp*. lacustre*, *Ptilimnium nodosum*, *Sidalcea nelsoniana*, and *Ziziphus celata* had low proportions of both germinating and vital staining, but had relatively high seed fill and exhibited evidence of insect predation or shriveled embryos. Germination proportions of *Kalmia buxifolia* and *Kalmiopsis fragrans* seeds were typical of the recently harvested cohort (0.56 and 0.53, respectively), but portions of positive staining were anomalously low (0.15 and 0.25); this was likely because these seeds were quite small (0.025 and 0.011 mg/seed), which made visualization of the staining pattern difficult.

**Table 4:** A summary of treatments given at sowing or during incubation that elicited the highest or lowest germination proportion and speed for species in the study group. Proportion data in this table are used in summary graphs provided in Figures 1 or 5. Time course data in Figures 2, 3, 4 are representative of data used to ascribe positive and negative effects of treatments on germination proportion.

Taxon	Proportion of germinating seeds^a^	Germination temperature (°C)	Germination speed^‐1^ (time to ⅔ total, days)	Response to removing outer coverings^b^	Response to GA_3_ b	Time at 5°C (days)^c^	Response to 5°C^b^	Response to 30 or 35°C^b^	After‐ripening or aging^d^	Proportion of positive staining seeds in TZ tests^a^, ^e^	Proportion of empty seeds^f^
*Abies fraseri*	0.00									0.05	0.96
*Abronia umbellata* subsp*. breviflora*	0.91	25	16	+		0	‐		+	nd	0.02
*Actaea arizonica*	0.03		155		( + )	114				0.40	0.04
*Agalinis densiflora*	0.90	20/30 or 20	299		( + )	35	+			0.78	0.01
*Aletes humilis*	0.16	5	357	‐	‐	126	+	‐		0.44	0.25
*Amaranthus pumilus*	1.00	20/30	118		(‐)	117	+		‐	0.60	0.00
*Amelanchier nantucketensis*	0.83	20	280	‐	‐	221	+		+	0.70	0.09
*Amorpha herbacea* var*. crenulata*	0.97	20/30	11	‐	‐	0	‐		‐	nd	0.00
*Amsonia tharpii* 1	0.90	20/30 or 25/35	8			0	‐	+	‐	nd	0.02
*Anemone patens* var*. multifida*	0.90	25	152			134	+		‐	1.00	0.08
*Arctostaphylos catalinae*	0.52	15	62	+	+	0			+	0.72	0.22
*Argemone glauca*	0.96	15/25	29		++	0				nd	0.04
*Astragalus albens*	1.00	20/30	11	++		0				nd	0.00
*Astragalus bibullatus*	0.85	20/30	4	++	(‐)	0	‐		‐	nd	0.00
*Astragalus rafaelensis* 2	0.72	25	5	++		0				0.67	0.01
*Astragalus magdalenae* var*. peirsonii*	1.00	20/30 or 25	57^4^	+		0				0.95	0.00
*Astragalus tegetarioides*	0.93	25	38	++		31		+		nd	0.00
*Astragalus tyghensis*	0.97	15/25	22	++		0			‐	0.97	0.00
*Berberis nevinii*	0.96	20 then 5	100	( + )	( + )	57	+^7^		+	1.00	0.00
*Besseya bullii* 1	0.97	15/25 or 25	12			0				nd	0.00
*Bidens torta* 1, 2	0.76	15/25 or 20/30	19			0	‐			0.30	0.12
*Boechera parishii*	0.96	5 to 20	7			26	+			nd	0.00
*Bromus carinatus* var*. carinatus*	0.98	20/30	14			10	( + )		+	0.94	0.00
*Calochortus umpquaensis*	1.00	5	83			131	+	‐	( + )	nd	0.00
*Campanula scabrella*	0.77	15/25 or 20	181		( + )	61	+		‐	0.77	0.03
*Carex comosa*	0.83	5	123		( + )	202	+		+	0.69	0.09
*Carex oronensis*	0.85	20/30	86		( + )	81	+			nd	0.00
*Castela emoryi*	0.62	20/30 or 20	85	( + )	‐	20	‐		+^5^	0.81	0.23
*Castilleja kaibabensis*	0.97	5 or 15/25	285		( + )	260	+		‐	1.00	0.01
*Ceanothus cyaneus*	0.62	20/30	91	+		90	+			0.77	0.26
*Chenopodium oahuense* 1	0.30	20/30 or 15/25	16			0				0.21	0.52
*Chrysopsis floridana* 1	0.24	20 or 20/30	20		( + )	0				0.25	0.71
*Cimicifuga elata*	0.84	20 then 5	94			231	+^7^		++	0.77	0.00
*Cirsium pitcheri*	0.46	15/25 or 15	97	( + )		114	+			0.80	0.23
*Clarkia biloba* subsp*. australis* 1	1.00	15	2			0			‐	nd	0.00
*Clematis socialis*	0.28	20 or 20/30	212	+	( + )	111	+		‐	0.82	0.39
*Clermontia kakeana*	0.70	20/30	85		( + )	0			+	nd	0.11
*Cordylanthus maritimus* subsp*. palustris*	0.78	15/25	66			60	+			0.82	0.03
*Cyanea angustifolia*	0.73	20/30	47			0	‐		‐	nd	0.10
*Cyperus javanicus* 1, 3	0.95	20/30 or 15/25	16			0			‐	nd	0.00
*Dalea foliosa*	0.99	15/25	32^4^	++		0				nd	0.00
*Deinandra increscens* subsp*. villosa*	0.82	5 or 20/30	51		( + )	23	+^6^			nd	0.06
*Deinandra mohavensis* 1	0.53	20/30	50			0			‐	0.67	0.21
*Dicerandra immaculata* 1	0.08	20/30 or 15/25	40			34				0.07	0.73
*Dodonaea viscosa*	0.95	20 or 15/25	9	++		0		‐		nd	0.00
*Dubautia menziesii*	0.03		38			0				nd	0.75
*Echinacea tennesseensis*	0.76	20/30 or 15/25	113		( + )	62	+		‐	0.47	0.22
*Echinocactus horizonthalonius* var*. nicholii* 3	0.85	20/30	42	+		0				1.00	0.00
*Erigeron parishii*	0.88	5 to 20	15			19	+			nd	0.03
*Eriogonum crosbyae* 8	0.00					3				nd	0.00
*Eriogonum cusickii* 1	0.78	20/30	40			0			‐	0.97	0.04
*Eryngium aristulatum* var*. parishii*	0.74	5 or 15	172	( + )	( + )	74	+	‐		0.60	0.17
*Eustachys petraea*	0.70	20/30 or 25	25		+	0			‐	0.74	0.31
*Eurybia furcata*	0.63	20	105			49	+			0.67	0.24
*Eutrema penlandii*	0.87	16	16		++	0				nd	0.03
*Gentiana newberryi*	0.59	15	118			110	+		‐	0.69	0.01
*Geum geniculatum*	0.94	15 or 20	106		( + )	98	+		‐	nd	0.01
*Gilia leptantha* subsp*. leptantha*	0.43	5 or 20	14		+	340	+			0.94	0
*Hedeoma diffusa*	0.58	25	69			68	+		‐	0.57	0.35
*Helonias bullata* 1	0.74	20/30	11			0				0.73	0.04
*Hesperocyparis forbesii*	0.43	20/30 or 25	37			26	+			0.39	0.18
*Hibiscus dasycalyx* 2	0.75	20/30	18	(‐)		11				0.55	0.22
*Horkelia hendersonii*	0.36	25	89		( + )	57	+^7^		( + )	0.69	0.34
*Hymenoxys texana* 1	0.96	25	3			0	‐			nd	0.02
*Kalmiopsis fragrans*	0.49	5 or 25	87			60	+^7^			0.25	0.10
*Kalmia buxifolia*	0.56	15/25 or 25	117			99				0.15	0.00
*Liatris novae‐angliae* 1, 8	0.78	20/30 or 25	32			0		+		nd	0.22
*Lilium parryi*	1.00	5 or 20	72			73	+		(‐)	nd	0.00
*Linum carteri* var*. carteri* 1	0.96	20/30	23			61	‐			0.11	0.00
*Lomatium bradshawii*	0.87	5 or 20/30	201		+	360	+	‐	+	nd	0.00
*Lupinus westianus* var*. aridorum*	0.85	20/30	17	++		0	(‐)			0.90	0.00
*Lycium sandwicense* 1	0.67	20/30	7			0				nd	0.22
*Metrosideros polymorpha* var*. polymorpha*	0.25	20/30 or 25	15		+	0				0.21	0.39
*Muhlenbergia microsperma*	0.56	20/30	133		++	20	( + )			1.00	0.00
*Nolina brittoniana* 1	0.86	20/30	29			0				nd	0.00
*Ornithostaphylos oppositifolia*	0.50	20	45	( + )	( + )	31	+		‐	0.78	0.12
*Osteomeles anthyllidifolia*	0.53	20/30	79	++		0				0.89	0.16
*Oxypolis canbyi*	0.03		150			68				0.40	0.39
*Packera franciscana* 2	0.12	15	57			36	+			0.10	0.52
*Penstemon clutei*	0.71	20	71			78	( + )	‐	‐	0.65	0.20
*Penstemon peckii*	0.87	20/30	3		+	0				nd	0.07
*Penstemon shastensis* 1	0.83	20/30 or 15/25	8			0			‐	0.50	0.13
*Phacelia formosula*	0.96	5 or 20	107		+	108	( + )^7^			nd	0.00
*Physaria globosa*	0.90	25 or 15/25	63		++	44			+	nd	0.00
*Physaria obcordata*	0.80	20/30	7		++	0			‐	nd	0.00
*Pinus radiata* 8	0.92	15/25	14	+		0		+		nd	0.00
*Pityopsis ruthii*	0.95	5 to 20	13			27			‐	nd	0.03
*Plagiobothrys hirtus*	1.00	20 then 5	221		+	102	+^7^		+	nd	0.00
*Polemonium eddyense*	0.76	15	126	+	( + )	62	+		+	0.97	0.00
*Polemonium occidentale* subsp*. lacustre* 1, 2	0.57	20/30	19			0				0.50	0.32
*Polyscias racemosa* 1	0.87	15/25	29			0			‐	0.80	0.02
*Ptilimnium nodosum*	0.17	20 or 15/25	98			62	+			0.20	0.10
*Purshia subintegra*	0.80	20/30	30			25	+		‐	nd	0.03
*Remirea maritima* 1	0.44	25/35 or 20/30	31			0		+	‐	0.40	0.53
*Remya kauaiensis*	0.00									nd	0.94
*Rhus kearneyi* subsp*. kearneyi*	0.52	20/30 or 25	35	++		10	+	‐	+	0.22	0.25
*Sarracenia oreophila*	0.78	20/30	62			20	+		‐	0.80	0.05
*Schoenoplectus tabernaemontani*	0.79	20/30	43		+	30	+			0.60	0.10
*Senecio ertterae*	0.39	25 or 5	71		+	59	+		+	0.50	0.30
*Sesbania tomentosa*	0.98	15/25	21	++		0				nd	0.00
*Sidalcea nelsoniana* 2	0.83	25	138	+		55	+			0.90	0.07
*Sisyrinchium sarmentosum*	0.61	15/25 or 20/30	593		( + )	234	+^7^		++	1.00	0.00
*Solidago plumosa* 1	0.93	20/30	5			0			‐	nd	0.07
*Tephrosia angustissima* var*. corallicola*	0.99	20/30	3	++		0				nd	0.00
*Vaccinium boreale*	0.70	15/25 or 20	79		( + )	0	+		‐	0.73	0.16
*Vaccinium crassifolium* subsp. *sempervirens*	0.53	20	131		( + )	232	+		‐	0.36	0.10
*Warea amplexifolia*	0.88	20/30	7		++	0			+	0.83	0.00
*Ziziphus celata* 1	0.22	25 or 20/30	42			10	‐			0.43	0.12
**No. of species with germination proportion **>**0.05**	**102**	**totals**	++	12	5		0	0	2		
			+	7	10		40	6	14		
			( + )	9	25		3	0	1		
			(‐) or ‐	1	1		7	7	31		

^a^ Proportion of germinating or TZ+ seeds: we report the value observed for either the recently harvested or stored cohort, whichever is larger. If a “+” is indicated in the after‐ripening column, the germination proportion is reported for the stored cohort. If the seed quality is low for the recently harvested cohort^2^, we report values for the stored cohort.

^b^ Responses are indicated by ++ (germination spikes immediately after treatment), + (germination increases following treatment but further testing needed to confirm), ( + ) (ambiguous results due to treatments delivered simultaneously or varying results between assays), (‐) (germination appears lower or slowed with treatment), and ‐ (lower germination following the treatment). A blank value indicates a treatment was not given or had no perceived effect.

^c^ Duration a sample was exposed to 5°C giving highest germination proportion. A value of 0 and a blank in the next column indicates a 5°C treatment was not given. A value of 0 and a “‐” in the next column indicates lower germination if the sample was exposed to 5°C compared to other treatments. A value >0 with a + in the next column indicates that germination increased following the 5°C treatment or germination occurred at 5°C. The duration at 5°C is often longer than the time to germinate (column 4) if the sample germinates at 5°C. Data represent exposure times delivered to the freshly harvested cohort; shorter durations may be feasible, but were not investigated.

^d^ Compares germination proportion and speed between recently harvested and stored cohorts and indicates after‐ripening for higher germination and speed (++) or germination speed (+) and aging for reduced germination proportion (‐). Blanks indicate that germination proportion for the two cohorts was within 0.2 and germination time did not differ by 30%.

^e^ nd means not determined. TTC assays were not conducted on samples with limited seed numbers, when germination was high in both cohorts, or when seed size was too small to visualize staining patterns.

^f^ Seed fill is reported for the cohort giving the higher germination proportion.

^1^ No particular cue is needed for seeds of these species to germinate, just water, the indicated germination temperature, and adequate time (indicated). Low germination may be due to low seed fill, damaged seed, or an undiagnosed problem (22 species).

^2^ The initial seed quality of the recently harvested seed was much lower than the quality of the stored counterpart in terms of embryo development or insect predation. This resulted in more and faster germination in the stored compared to recently harvested sample—the criteria used to indicate after‐ripening effects.

^3^ The initial seed quality of the stored sample was low due to poor embryo development. This resulted in the appearance of a large difference in germination proportion, but this was probably not due to aging.

^4^ These species were clipped at various times after sowing to demonstrate an immediate response to clipping. Germination times reflect time after sowing. Time after clipping is six (*Astragalus magdalenae* var*. peirsonii*) and two (*Dalea foliosa*) (Figure 4A) days.

^5^ Indications of after‐ripening; germination is faster but lower in stored seeds (*Castela emoryi*).

^6^ Germination occurs at both 5°C and 20/30°C but is faster at 5°C (Figure 3D).

^7^ Warmth followed by cold gave the highest germination.

^8^ Recently harvested seeds were not available and so only the stored seeds were tested.

Results of the TZ assays were useful in identifying seeds that were alive but slow to germinate, and this prompted additional treatments and prolonged germination assays. We characterized germination speed as the number of days required for the germination proportion to reach 67% of its final value. The average time was 74 ± 87 days (*n* = 102) (Table 3) and ranged from two (*Clarkia biloba* subsp*. australis*) to nearly 600 days *(Sisyrinchium sarmentosum*) after sowing (Figure 1C, Table 4). For perspective, observations at four and seven days are often recommended for seed tests of domesticated species, and standardized tests rarely exceed 30 days (AOSA, 2018). Samples with low seed fill or unbroken dormancy are indicated in Figure 1C to avoid conflating these anomalies with fast germination. About one‐fourth of the species in this study produced seeds that germinated quickly (≤15 days, 24 species), about one‐half of the seeds germinated at moderate speeds (between 16 and 90 days, 47 species), and nearly one‐third of the seeds germinated slowly (>90 days, 31 species) (Figure 1C, Table 4). Most of the seeds with water‐impermeable (i.e., “hard”) seed coats germinated quickly if the seed coat was clipped before or soon after sowing.

**Figure 2:**
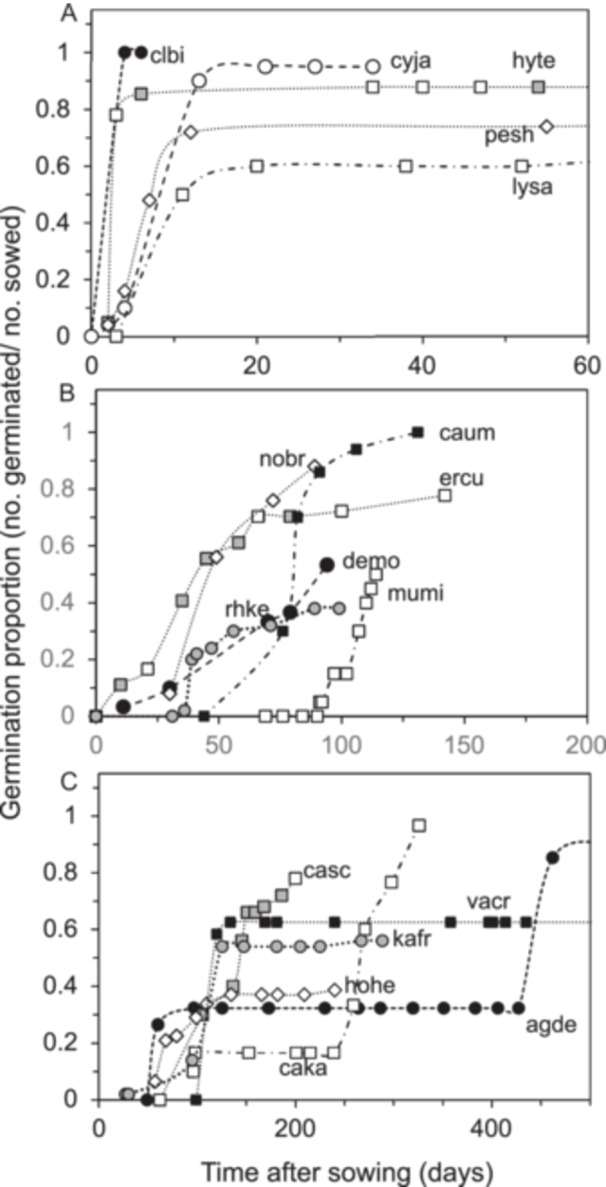
Germination time courses of recently harvested seeds that are representative of fast (A), moderate (B), and slow (C) germination. In B and C, abrupt increases in germination usually occur when a temperature or hydration treatment relaxes seed dormancy (Table 4). The different symbols distinguish each species on the graphs, and letter codes are provided in Table 2.

The time required for seeds to germinate can provide insights into germination requirements and synchrony. We developed germination time courses to characterize germination speed and link abrupt increases in germination to a particular treatment (Figure 2). Representative time courses of seeds that germinated quickly (≤15 days) showed that radicles often began to emerge within four days after sowing and that germination was mostly completed by 20 days (Figure 2A). The average germination time was 8 ± 4 days for this subset of 24 species, and germination proportions were higher than the overall average (0.79 ± 0.23); ungerminated seeds usually lacked embryos. We considered this group of seeds to be ready‐to‐germinate, with delays only occurring if water‐impermeable seed coverings were not scarified. In contrast, germination of slow‐germinating seeds (>90 days) often occurred as abrupt steps following a temperature transfer or weakening of water‐permeable seed coverings (Figure 2C). The average germination time was 168 ± 104 days for this subset of 31 species, and germination proportions were slightly lower than the overall average (0.60 ± 0.28). Samples that germinated at moderate speeds (between 16 and 90 days, average time was 45 ± 23 days among the 47 species) exhibited a combination of stepwise or asynchronous patterns that often reflected brief exposure to 5°C or several rehydration cycles with GA_3_ solution (Figure 2B). The average germination proportion for this subset reflected the overall average of the cohort (0.67 ± 0.27).

### Treatments affecting germination proportion or speed in recently harvested samples

Temperature is the most important factor affecting germination response, and we tried to optimize this treatment if there was a sufficient number of seeds in the sample. Initial conditions with daily cycles of 20°C and 30°C or 15°C and 25°C produced the highest germination in 49 and 18 species, respectively (out of 103, with or without a prior 5°C exposure), although some of these species germinated equally well at a range of temperatures (Table 4). Constant 5°C or 15°C treatments promoted the highest germination proportions in 11 and eight species, respectively. Only 22 species germinated in the presence of just water and warmth, usually at alternating temperatures (Table 4).

#### Low and high temperature treatments

**Figure 3:**
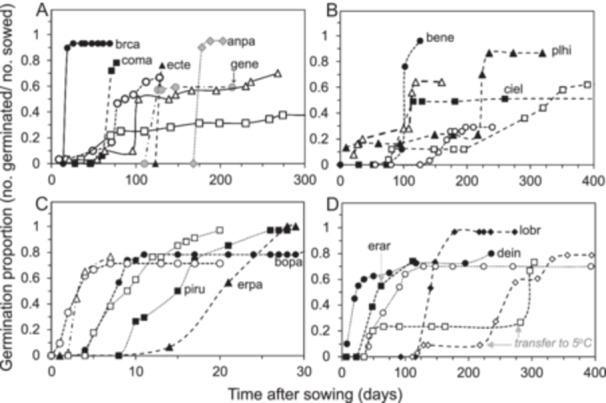
Time courses showing stimulated germination by 5°C treatments in recently harvested seeds: 5°C treatment before transfer to warmth (A), 5°C treatment after incubation in warmth (B), constant 5°C treatment of fast‐germinating seeds that also germinate at warmer temperatures (C), and constant 5°C treatment of slow‐germinating seeds (D). Germination is stimulated (A, B, D) (black points) by the duration and timing of the 5°C treatment compared to no 5°C treatment (white points); there are no contrasting treatments to validate 5°C stimulation (gray points). Abbreviations representing the species names are provided in Table 2, and germination speeds are listed in Table 4.

Stratification (exposure of hydrated seeds to cold [5°C] before transfer to warmth) stimulated germination in more samples than any other treatment, with 43 of 101 species presenting the highest total germination during or following exposure to 5°C for as little as 10 and up to 365 days (Figure 3). Germination was lower, slower, and asynchronous in numerous species that were placed at warm temperatures without an initial 5°C exposure (Figure 3A, compare germination speed for species marked with black vs. white symbols; species marked with gray symbols show full germination a few days after transfer from 5°C, but lack a stratification control). Reversing the order for stratification (i.e., transferring from warm to cold temperatures) was noted to stimulate germination in a few species, as has been reported for congeners (Baskin and Baskin, 2014) (Figure 3B, Table 4). A few species with broad temperature optima germinated within two weeks at a constant temperature of 5°C, but these seeds germinated faster, albeit at slightly lower proportions, at warmer temperatures (Figure 3C, compare germination speed for species marked with black vs. white symbol pairs). We found that maintaining some seeds at 5°C, with no intermittent warmth, achieved the highest germination proportions even if it took many days for radicle emergence (Figure 3D, see also Figure 2B [caum] and 2C [caka and kafr] [species abbreviations are provided in Table 2]; this is also the case for *Lilium parryi* [not shown]).

An alternative temperature treatment is heat shock, which exposes seeds to 35°C. Heat shock appears to stimulate germination of seeds like papaya and cuphea that originate from tropical climates (Wood et al., 2000; Crane et al., 2003). We found that germination was faster in seeds of *Amsonia tharpii*, *Astragalus tegetarioides*, *Liatris novae‐angliae*, *Pinus radiata*, and *Remirea maritima* if they were initially placed in an incubator at alternating temperatures of 25°C and 35°C (Table 4, time courses not shown).

#### Scarification, GA_3_, after‐ripening, and treatments that damaged seeds

**Figure 4:**
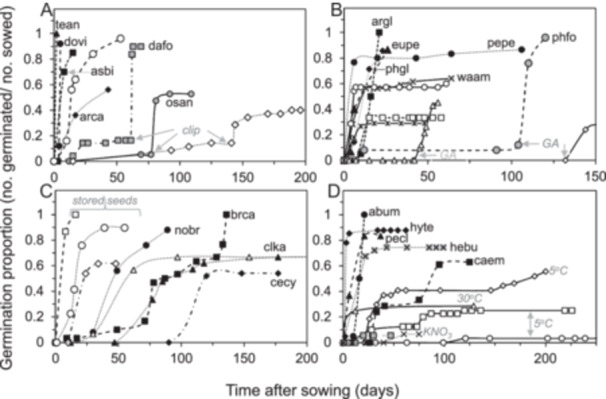
Time courses showing germination stimulated by treatments that did not involve exposure to 5°C (A, B, C) and treatments that inadvertently inhibited germination (D). Treatments considered were scarification (A), hydration or rehydration with GA_3_ (B), and storage to induce after‐ripening (C). Stimulation of germination by treatment is represented by black points, and the non‐treatment control is represented white points (Table 4). Abbreviations representing the species names are provided in Table 2.

Several other treatments, in addition to temperature, elicited germination responses. Clipping seeds that had water‐impermeable seed coverings allowed them to imbibe and led to rapid germination (Figure 4A). Of the 19 or so species exhibiting “hard” seeds, most maintained germination potential in the presence of water and germinated fully once scarified. In contrast, seed coverings of *Arctostaphylos catalinae* (Figure 4A), *Clematis socialis*, and *Rhus kearneyi* subsp*. kearneyi* (not shown) allowed some water uptake, and delayed scarification reduced final germination in these species. Scarification appeared to hasten germination of some species that were not exposed to 5°C for a sufficient time (e.g., ecte [Figure 3A] and bene [Figure 3B]).

The phytohormone GA_3_ is known to stimulate germination for many species (AOSA, 2018). Initially hydrating seeds with GA_3_ solutions or rehydrating seeds during incubation led to faster and higher germination rates than water alone in numerous species, especially those within Brassicaeae and two of the three grass species studied (Figure 4B, also compare black and white symbols of same shape and upswing of agde in Figure 2C). In several cases, stimulatory effects of GA_3_ are ambiguous because it was delivered simultaneously with scarification or stratification upon sowing seeds and there were too few seeds in the sample to test the treatments separately (indicated by (+) in Table 4).

Dry after‐ripening plays a role in relaxing dormancy of seeds from some species that resist germination when sowed soon after harvest but readily germinate after a few months of storage at ambient temperatures or several years of storage at low temperatures (Gianinetti and Cohn, 2007; Bazin et al., 2011; Von Mark et al., 2013; Nelson et al., 2023). Germination was faster and proportions sometimes higher in the stored cohorts of several species compared to their recently harvested counterparts that were classified as germinating slowly or at moderate speed (Figure 4C, compare white symbols [stored] with black symbols of same shape). Examples not shown in Figure 4C are *Amelanchier nantucketensis*, *Castilleja kaibabensis* (Figure 2C), *Plagiobothrys hirtus* (Figure 3B), and *Sisyrinchium sarmentosum*, all species producing slow‐germinating seeds that require extensive time at 5°C to germinate; this time was nearly halved in the stored seeds.

In our efforts to stimulate germination, we sometimes encountered treatments that were clearly detrimental. Germination proportions were reduced by prolonged exposure to 5°C (e.g., *Abronia umbellata* subsp. *breviflora* [see also Kaye, 1999], *Hymenoxys texana*, and *Castela emoryi* [Figure 4D], as well as *Ornithostaphylos oppositifolia* and *Cyanea angustifolia* [Table 4]). On the other hand, exposure of some seeds to 30°C in a 20/30°C daily cycle drastically reduced germination compared to a 15/25°C cycle or constant 15°C or 25°C treatments (e.g., *Penstemon clutei* [Figure 4D], *Dodonaea viscosa*, *Rhus kearneyi* subsp. *kearneyi*, and possibly *Besseya bullii* and *Plagiobothrys hirtus* [time courses not shown, see Table 4]), and germination was lower in *Calochortus umpquaensis* and some species within Apiaceae if exposed to 20°C or 25°C. Premature clipping or removal of outer seed coverings introduced fungal contamination in some species (e.g., *Berberis nevinii*, *Hibiscus dasycalyx*, and *Osteomeles anthyllidifolia*) (Table 4). Genebanks sometimes use a dilute solution of KNO_3_ to stimulate germination, but *Helonias bullata* seeds did not germinate in the presence of KNO_3_ (Figure 4D), and transferring seeds to water increased germination. Similarly, germination was lower in seeds of *Astragalus bibullatus* and *Penstemon clutei* that were exposed to GA_3_, compared to hydration with water alone.

### Effect of storage on seed viability and germination time (speed)

**Figure 5:**
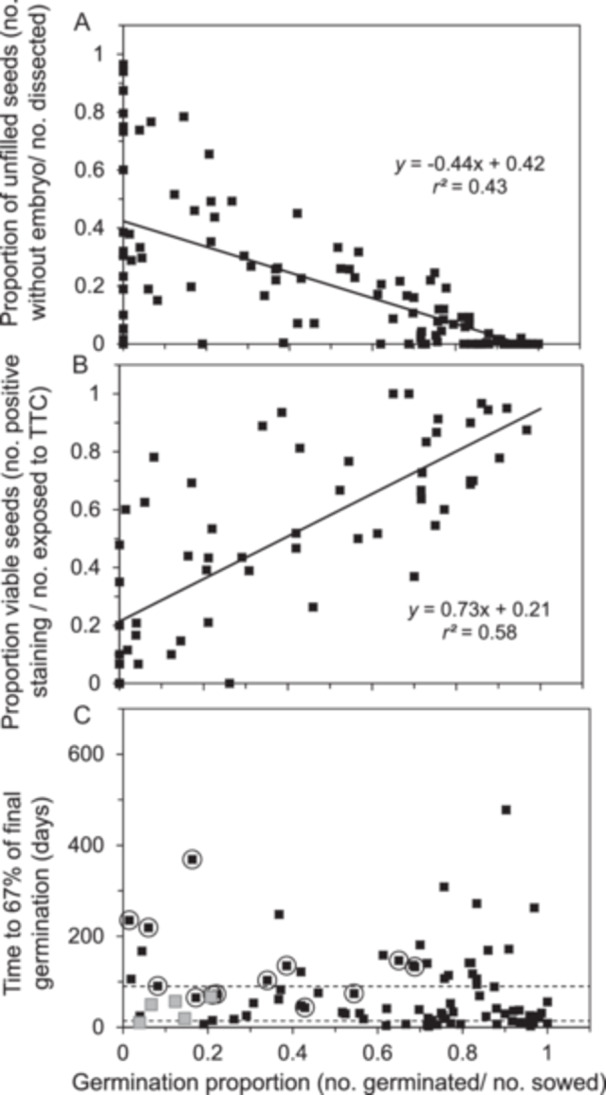
Relationships between various measurements of viability and vigor in stored seeds from 108 species. Seeds were stored at −18°C for 28 ± 7 years. The germination proportion negatively correlates with the proportion of empty seeds (A) and positively correlates with respiratory activity determined using TTC staining patterns (B), although relationships appear more scattered in stored seeds compared to recently harvested cohorts (Figure 1). The germination proportion does not correlate with germination speed (C). In C, gray points represent samples with a high proportion of empty seeds (>0.5) and encircled points represent samples with unbroken dormancy.

As noted previously, storage may stimulate germination in some species as an after‐ripening effect. However, the predominant effects of storage were either negligible (most species) or detrimental (31 species), based on comparisons of germination proportion or speed between recently harvested and stored cohorts (Table 4). Average proportions of germination and positive staining in TZ tests (TZ+) in the stored cohort were 0.50 ± 0.36 and 0.50 ± 0.32, respectively, which were lower than the same metrics reported for recently harvested seeds (germination test: *t* = 5.10, *df* = 103, *P* « 0.01; TZ test: *t* = 2.45, *df* = 49, *P *< 0.02, *t*‐test of means using paired samples). Lower viability cannot be attributed to more unfilled seeds in the stored samples, as proportions of empty seeds were similar between cohorts (Table 3; *t* = −0.76, *df* = 103, *P* » 0.10, *t*‐test of means using paired samples). Seed fill also negatively correlated with germination proportion in the stored cohort (Figure 5A; *F* = 81, *df* = 106, *P* « 0.01), but the weaker correlation (*r* ^2^ = 0.43) and shallower slope, compared to recently harvested seeds, indicated that other factors contributed to the variation of germination proportion, namely death by aging. We call attention to the greater number of points with germination proportion <0.1 (Figure 5A, left side of graph) compared to the recently harvested cohort (Figure 1A), as well as the cluster of points with germination proportion >0.8 (Figure 5A, right side of graph), collectively indicating different responses to storage among the samples. Viability assessed by positive staining in the TZ test also correlated with germination proportion in stored seeds (Figure 5B; *F* = 77, *df* = 57, *P* « 0.01). Germination is lower than positive staining in several species and should not be attributed to dormancy because germination requirements were resolved in experiments using the recently harvested cohort. This observation, and the weaker correlation coefficient and slope (Figure 5B) compared to a similar correlation in the recently harvested cohort (Figure 1B), suggests the staining patterns of the TZ tests may be less sensitive to storage time than germination assays.

In addition to viability, stored seeds tended to germinate slower than their recently harvested counterparts (Figure 5C). Time to germinate increased from an overall average of 74 to 81 days for recently harvested and stored seeds (Table 3), which was not a significant difference considering the overall variation in germination speed. The effect of storage was mostly manifested in seeds that were classified as ready‐to‐germinate; the average days to germinate increased to 23 ± 28 days (*t* = −2.63, *df* = 21, *P *< 0.01, test of means using paired samples). For seeds that germinated at moderate speeds in the recent cohort, the average days to germinate increased to 75 ± 70 days, *t* = −2.70, *df* = 39, *P *< 0.01, test of means using paired samples). Owing to mixed results of dry after‐ripening and aging, germination speeds for the slowest germinating seeds were comparable between cohorts (*t* = 1.13, *df* = 22, *P* > 0.10, test of means using paired samples).

## DISCUSSION

### Widely varying germination requirements challenge genebank standardization

This paper examines viability and viability testing methods in wild‐collected seeds. Seed germination provides critical information about the ability of seeds to develop normal roots and shoots, and standardized methods that elicit healthy seedling growth facilitate comparisons among seed lots, testing labs, and storage times.

The effect of common testing conditions on the germination of seeds from many wild species is largely unknown. Hence, development of seed testing protocols often begins with understanding germination cues, which can vary with the ecology or phylogeny of wild species (Fuller and Allaby, 2009; Baskin and Baskin, 2014; AOSA, 2018; Kildisheva et al., 2020). Instead of germination tests, seed analysts may use tests that imply viability, such as respiratory capacity of embryonic tissues using staining patterns of a TZ test (Copeland and McDonald, 2001; Miller, 2005; Riebkes et al., 2015). A TZ test takes about two days, uses standardizable approaches, and circumvents the need to stimulate and optimize germination. However, surrogate assays do not inform about the conditions that will be needed to *use* germplasm in a grow‐out or restoration effort. Moreover, viability assays that preclude the observation of tissue growth and development might not foretell whether germplasm was damaged during preservation. In parallel efforts preserving tissue‐cultured explants, normal plant growth is the standard criterion to evaluate successful treatments, and this requires developing applicable growth conditions—often at the genotype level—before attempting preservation (Nagel et al., 2024). In this context, genebanks are charged with demonstrating that surrogate viability assessments accurately reflect germplasm capacity to grow normally before and after preservation. In other words, genebanks are challenged to balance the problem of managing diversity, which will include diverse responses to stress and requirements for growth, with the necessity of using standardized preservation treatments and testing approaches.

**Figure 6:**
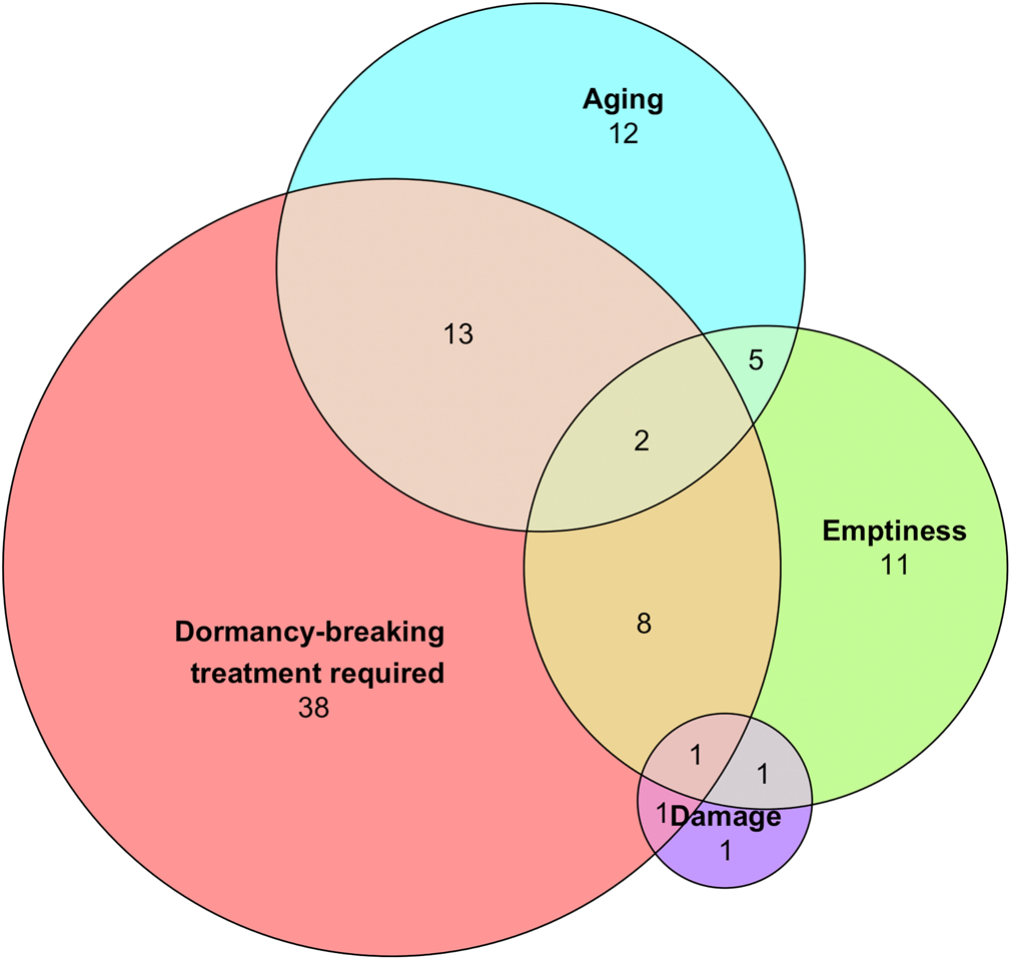
A Venn diagram depicting the effect of various D.E.A.D. factors (dormant, empty, aged, and damaged) on the germination results characterized for the 108 wild species in this study. Seeds from more than one‐half of the species required a dormancy‐breaking treatment to stimulate germination. Roughly one‐fourth had low seed fill (>33% empty), and roughly one‐third exhibited evidence of aging in the stored cohort. Incidence of damaged seeds (herbivory, immature harvest, or fungal infestation) was relatively low in this dataset.

This study illustrates the genebank dilemma of balancing the need to accommodate diversity with standardization. The study focused on the assessment of viability in seeds from over 100 wild species from diverse habitats, growth habits, and botanical backgrounds. Standardized tests are facilitated by the rapid, synchronous, and thorough germination that is characteristic of seeds from domesticated species (AOSA, 2018). About one‐fourth of the recently harvested samples exhibited these ready‐to‐germinate characteristics, with radicle emergence apparent within 2–4 days and the assay complete (>0.80 germination) within 15 days (Figures 1C, 2A). In contrast, more than three‐fourths of samples exhibited some form of dormancy or resistance to germination (Figures 2B, 2C, 6); several of these samples were extremely slow to germinate (Figures 1C, 2C), suggesting “deep” (sensu Baskin and Baskin, 2014) dormancy or complex dormancy‐breaking treatments. The positive correlation between germination and TTC staining patterns (Figure 1B) was weaker than expected (*r* ^2^ = 0.64) considering the intense efforts to elicit a germination response (Figures 2, 3), and perhaps indicates that uncontrolled factors besides dormancy contribute to both viability metrics.

### Genebanks and samples with low viability

Failure to germinate could be an indicator of dormancy, as described above, or a symptom of death. It may be surprising to note that there are few symptoms to help us distinguish dormancy from death. Turgidity is frequently used to identify seeds that are not dead yet, if all the seed structures are present and normal. Assays that quantify the metabolic capacity of hydrated seeds (Bello and Bradford, 2016; Dalziell and Tomlinson, 2017; Fleming et al., 2021) or RNA integrity in dry seeds (Fleming et al., 2019; Tetreault et al., 2025; Walters et al., unpublished) may soon be available to indicate low viability. Another important indicator of low viability is the presence of anomalous embryonic structures, which can be visualized by dissecting seeds, as we did in this work, or non‐invasively using X‐rays (Riebkes et al., 2015). In this study, the absence of embryos (i.e., empty seeds) was a prevalent structural anomaly that varied widely among samples and negatively correlated with germination capacity, accounting for nearly 70% of the variation in germination proportion among recently harvested seeds (Figures 1A, 6).

Structural anomalies in seeds usually occur before seeds arrive at the genebank, often in response to stress during seed development or harvest. A high incidence of empty seeds may indicate pollen limitation and declining pollinators (Knight et al., 2005). Alternatively, empty seeds may arise when the embryogenic program finishes and mature seeds shatter, a common feature of wild plants that is lost upon domestication (Liu et al., 2018). Embryos may also be misshapen, shriveled, or nicked because of microbial or insect infestation, premature harvest, improper drying, or overzealous thrashing. The low incidence of damaged embryos among samples in this study suggests well‐timed harvests and gentle handling (Figure 6).

Widely ranging viability, as we observed in recently harvested samples, is another consequence of diversity that challenges the need for standardization in genebanking. Currently, international standards set the benchmark for sample viability at 0.85 to ensure that genebanked samples are genetically representative of their source populations (FAO, 2014; MSBP, 2015; CPC, 2019; De Vitis et al., 2020); this is a high bar, according to which at least 33 samples in this study would be identified as substandard due to >0.15 of samples being empty. However, low viability due to seed fill or insect predation does not appear to affect genebanking responses of the remaining viable seeds, and so while it is appropriate to acknowledge samples with these kinds of embryo anomalies, they should not be discarded (Mead and Gray, 1999). In this context, genebank management of empty seeds becomes a matter of practicality in terms of the resources needed to clean, package, and store the seeds.

Implicit in the discussion of viability standards for genebanked samples is the recognition that viability declines during storage and mortality should not exceed 15% of the original viability (FAO, 2014). In other words, seed deaths due to time‐dependent damage (i.e., aging) can also explain why seeds fail to germinate. The rate of seed aging for seeds stored at −18°C is mostly undocumented, but international standards based on a monitoring frequency of 15–20 years suggests that seed life expectancies between 25 and 50 years are expected (FAO, 2014). Seeds in this study were stored for 28 ± 7 years, and a major finding is that seeds in most of the samples survived this relatively long storage period (Table 3, Figure 5). However, there are indications that about one‐fourth of the stored samples lost viability due to aging (Figure 6, Table 4). The correlation coefficients (slopes and *r* ^2^ values) between proportions of germinating, empty, and viable seeds were lower for the stored cohort (Figure 5A, B) compared to the recently harvested cohort (Figure 1A, B), and we interpret this to mean that some of the variation is attributed to seed deaths by aging, which is not accounted for by missing embryos or staining patterns in TZ tests.

Aging is usually identified by seed deaths over time, probably because there are few other recognized symptoms. We report that germination is slower in seeds that are aging but still alive (Table 3, Figure 5C). The number of days for seeds to germinate increased most dramatically in the subset of recently harvested seeds that were fastest to germinate. A similar effect was also reported for stored crop seeds that usually are not dormant and germinate rapidly when fresh (Priestley, 1986; Powell and Matthews, 2012; Fleming et al., 2021). Delayed germination was less apparent in stored samples from the subset of deeply dormant seeds, which we attribute to the combined and opposing effects of after‐ripening or relaxed germination requirements (Figure 4C).

Delayed germination of dying seeds is a manifestation of aging that can be detected before the seed dies. Genebanks would benefit from additional tools that detect aging progress before seeds die and warn of imminent mortality and the need to use or regenerate the sample before it is lost to aging (Hay et al., 2025). New tests should use standardizable methods and yield quantifiable, calibrated data so that genebanks are less dependent on viability, which is relatively insensitive to time initially but then declines abruptly (De Vitis et al., 2020; Walters et al., 2005, 2020; Tetreault et al., 2023). New technologies should also be less dependent on large seed numbers. We are recommending supplementing germination testing with additional tests, rather than replacing germination tests altogether.

### Species characteristics and interactions between germination and aging patterns

In selecting the ~100 diverse species for this study, we hypothesized we would observe species‐specific variation in germination behaviors and seed aging rates, and we planned to use this variation in correlations with phylogenetic and ecological factors (Heineman et al., unpublished). The concept of species‐specific dormancy mechanisms and germination requirements has been considered in the context of biogeography for thousands of wild species around the globe (Fuller and Allaby, 2009; Baskin and Baskin, 2014; Kildisheva et al., 2020). In fact, we drew insights about possible dormancy‐breaking treatments based on germination patterns and seed testing protocols published for congeneric species (e.g., Baskin and Baskin, 2014; AOSA, 2018). Similarly, there are many foundational studies of crops and wild plants that report fast and slow aging seeds in soil, warehouse, and genebank contexts (Priestley, 1986; Walters et al., 2005; Probert et al., 2009; Fleming et al., 2023). Also supporting the hypothesis that seed aging rates are species‐specific are numerous studies that report species‐level constants for aging models (SID et al., 2023).

The expression of species‐specific seed germination and longevity characteristics possibly suggests there is an interaction between these traits. Both traits appear to be regulated by similar molecular mechanisms during the latter phases of embryogenesis (Ramtekey et al., 2022; Rehmani et al., 2022; Pirredda et al., 2023; Tan et al., 2025). Evidence that the delay‐of‐germination gene (Bentsink et al., 2006) also links to longer‐lived seeds in *Arabidopsis* also provides compelling evidence of a positive relationship. This paper presents evidence to the contrary: of 32 species in the subset of seeds that germinated fastest (i.e., not dormant), only nine appeared to be fast aging (Table 4), and there is no indication that slower‐germinating seeds (i.e., dormant) fared better during storage than the faster‐germinating seeds. However, results from this study appear to support the long‐held notion that seeds with physical dormancy (i.e., an impermeable seed coat) are long‐lived (Priestley, 1986; Baskin and Baskin, 2014; Ramtekey et al., 2022). Here, some level of deterioration was observed in seeds from 29 species, 25 of which had water‐permeable seed coverings. However, seeds with permeable seed coats do not appear to be short‐lived; 55 out of 79 species that showed little evidence of aging during storage also had seed coverings that were permeable to water (Table 4).

Despite the claims that seed traits are regulated at the species level, there is considerable variation in the expression of these traits within species. The wide and unexplained intraspecies variation in seed longevity makes it difficult to predict for a particular sample and is the reason that genebanks must monitor viability (Walters et al., 2005). Seed longevity is known as a complex trait because it is controlled by interacting genetic and environmental factors (Zinsmeister et al., 2020; Ramtekey et al., 2022; Tan et al., 2025). For example, both the embryogenic program and weather affect the rate of seed maturation, potentially leading to the collection of immature seeds, which tend to age faster than fully mature seeds (Hay and Probert, 1995; Righetti et al., 2015; Ramtekey et al., 2022; Pirredda et al., 2023).

## CONCLUSIONS

Genebanks must balance the opposing interests of handling diverse materials in standardized ways. This dilemma becomes especially challenging when working with seeds from wild species, which may range in sample quality because of uncontrolled growth conditions in natural environments as well as undocumented seed traits such as germination behavior and shelf life. Unlike seeds from domesticated species, which are fairly uniform and usually germinate quickly and synchronously, seeds from wild species may take days to years to germinate, so that it is often difficult to know whether an ungerminated seed is alive or not. We categorized the reasons that seeds do not germinate in terms of the acronym D.E.A.D. (dormant, empty, aged, or damaged) and then explored these factors in seeds from wild populations of endangered plant species across the United States (both recently collected seeds and seeds that had been stored for decades). We assessed the viability of samples using germination assays, tetrazolium staining patterns, and the presence of empty seeds. While tetrazolium and seed fill tests can be standardized, they do not reveal information about whether a genebanked seed is capable of normal growth. Hence, we attempted to break dormancy and elicit germination using various temperature and hydration treatments. Successful treatments varied among the 100 species included in the study. We advocate for new technologies that describe degradation before the decline of viability. Many of the seeds that were stored for decades germinated well using these treatments, providing direct evidence of the feasibility and promise of genebanking as an ex situ conservation strategy.

## AUTHOR CONTRIBUTIONS

C.W. is co‐Principal Investigator on the Institute of Museum and Library Services (IMLS) grant, analyzed the data, and co‐wrote the manuscript with L.M.H. L.M.H. coordinated laboratory activities, supervised data collection, and co‐wrote the manuscript. K.D.H. is Principal Investigator on the IMLS grant, coordinated seed collections among the Center for Plant Conservation (CPC) Botanical Garden collaborators, and contributed insights, analyses, and critical review/edit of the manuscript. H.M.T. contributed to the manuscript. S.I. and K.M. provided technical support and data and approved the contents of the manuscript. S.A., C.B., K.F., D.W., M.K., S.M., N.K., A.S., N.M., T.N.K., C.P., M.A.A., N.D., S.B., D.R, W.G., A.T., E.C., J.L., L.B., C.R., H.E.S., K.N., D.S., J.F., K.W., and R.J. advised on species selection for study, contributed botanical expertise, oversaw seed collections for recently harvested seeds as listed in Table 2 and distributions of stored seeds, and reviewed, edited, and approved the manuscript. J.M. is co‐Principal Investigator on the IMLS grant, provided leadership for Agricultural Research Service (ARS) and CPC interactions, and critically reviewed and edited the manuscript.

## Data Availability

The species included in this study and the institutions that donated materials are listed in Table 2. Table 4 includes the seed treatments that were used to stimulate germination, as well as the results of the seed quality assessments for each species studied, usually for the recently harvested sample. A future publication will provide germination data for the stored seeds as well as estimates of seed longevity for the species. Curatorial data for the accessions in this study are available by contacting the CPC National Office at info@saveplants.org.
